# Integration of Partial Least Squares Regression and Hyperspectral Data Processing for the Nondestructive Detection of the Scaling Rate of Carp (*Cyprinus carpio*)

**DOI:** 10.3390/foods9040500

**Published:** 2020-04-16

**Authors:** Huihui Wang, Kunlun Wang, Xinyu Zhu, Peng Zhang, Jixin Yang, Mingqian Tan

**Affiliations:** 1 School of Mechanical Engineering & Automation, Dalian Polytechnic University, Dalian 116034, China; whh419@126.com (H.W.); w1074224274@163.com (K.W.); Zxyznjc@163.com (X.Z.); zhangpeng@dlpu.edu.cn (P.Z.); yangjixin_0087@126.com (J.Y.); 2 School of Food Science and Technology, National Engineering Research Center of Seafood, Dalian Polytechnic University, Dalian 116034, China; 3 Collaborative Innovation Center of Seafood Deep Processing, Dalian 116034, China; 4 Engineering Research Center of Seafood of Ministry of Education of China, Dalian 116034, China

**Keywords:** carp, scaling rate, hyperspectral, partial least square regression, preprocessing

## Abstract

The scaling rate of carp is one of the most important factors restricting the automation and intelligence level of carp processing. In order to solve the shortcomings of the commonly-used manual detection, this paper aimed to study the potential of hyperspectral technology (400–1024.7 nm) in detecting the scaling rate of carp. The whole fish body was divided into three regions (belly, back, and tail) for analysis because spectral responses are different for different regions. Different preprocessing methods, including Savitzky–Golay (SG), first derivative (FD), multivariate scattering correction (MSC), and standard normal variate (SNV) were applied for spectrum pretreatment. Then, the successive projections algorithm (SPA), regression coefficient (RC), and two-dimensional correlation spectroscopy (2D-COS) were applied for selecting characteristic wavelengths (CWs), respectively. The partial least square regression (PLSR) models for scaling rate detection using full wavelengths (FWs) and CWs were established. According to the modeling results, FD-RC-PLSR, SNV-SPA-PLSR, and SNV-RC-PLSR were determined to be the optimal models for predicting the scaling rate in the back (the coefficient of determination in calibration set (*R_C_*^2^) = 96.23%, the coefficient of determination in prediction set (*R_P_*^2^) = 95.55%, root mean square error by calibration (*RMSEC*) = 6.20%, the root mean square error by prediction (*RMSEP*)= 7.54%, and the relative percent deviation (*RPD*) = 3.98), belly (*R_C_*^2^ = 93.44%, *R_P_*^2^ = 90.81%, *RMSEC* = 8.05%, *RMSEP* = 9.13%, and *RPD* = 3.07) and tail (*R_C_*^2^ = 95.34%, *R_P_*^2^ = 93.71%, *RMSEC* = 6.66%, *RMSEP* = 8.37%, and *RPD* = 3.42) regions, respectively. It can be seen that PLSR integrated with specific pretreatment and dimension reduction methods had great potential for scaling rate detection in different carp regions. These results confirmed the possibility of using hyperspectral technology in nondestructive and convenient detection of the scaling rate of carp.

## 1. Introduction

Carp (*Cyprinus carpio*) is a freshwater fish popular among the Chinese and other Asian populations. The production and consumption of carp have increased year by year. The investigation into fish deep processing enterprises revealed that the main processes of carp processing included scaling, gutting, cleaning, and freezing. Of these, scaling is an important procedure to ensure the shelf life and quality appearance of carp products, which directly affects their apparent quality and commercial value. Furthermore, the parameters of the scaling device can be adjusted according to the scaling rate to achieve a better processing effect. At present, the scaling rate of carp is mainly detected manually, which is highly subjective and seriously restricts the automation level of processing of this freshwater fish product. Therefore, developing a nondestructive, fast, and convenient automatic method for detecting the scaling rate of carp is essential.

Machine vision has been widely applied in the online detection of agricultural by-products with the advancement of computer science and sensing technology [1]. This technology combined with image processing, pattern recognition, and artificial intelligence technology can detect samples in a noncontact manner [2]. The color [3], size [4], shape [5], and other apparent features of the sample can be analyzed and processed from collected images for detection. However, some characteristics are difficult to detect because traditional machine vision is conducted at visible wavelengths [6].

Hyperspectral information has abundant data that combines images with spectra [7]. Besides reflecting the shape, characteristics [8], color [9], and texture [10] of samples, this information can also be used to analyze the physicochemical properties, such as sugar content [11], acidity [12], water content [13], and so forth. Many studies showed that hyperspectral data were more reliable than traditional machine vision or spectral technology [14,15]. At present, hyperspectral technology has been used in the detection of food freshness [16], quality classification [17], nutrient content [18], food adulteration [19], and fruit [20] and vegetable [21] surface damage. Furthermore, hyperspectral imaging technology has also been widely used in fish for detecting nematode contamination, determining physical and chemical properties, predicting chemical composition and freshness, and discriminating and measuring microbial spoilage [22,23]. While applying this technology, it is necessary to implement a preprocessing method to reduce the interference signal and highlight the characteristic information of the spectra. Savitzky–Golay (SG) [24], first derivative (FD) [25], multiple scattering correction (MSC) [26], and standard normal variate (SNV) [27] methods have been proven to be effective in spectrum pretreatment. It was shown that these methods could reduce the influence of external factors and improve the detection accuracy to some extent [28]. Furthermore, dimension reduction has become a necessary process due to the huge amount of hyperspectral data [29]. Many methods of dimension reduction for hyperspectral data have been established. Successive projection algorithm (SPA), regression coefficient (RC), and two-dimensional correlation spectroscopy (2D-COS) are popular methods used, which can eliminate the wavelengths with high correlation, reduce the complexity of the calculation, and improve the precision of models [30,31].

The purpose of this study was to apply hyperspectral technology to realize the nondestructive detection of the scaling rate of carp. The hyperspectral imaging system was set up to obtain the spectra of each image pixel in the visible and near-infrared wavelength range (387.1–1024.7 nm). The specific objectives of the study were to: (1) realize the spectral preprocessing and characteristic wavelength (CW) selection using different methods; (2) establish the scaling rate detection model using full wavelength (FW) and CW; (3) select the most suitable model for scaling rate detection.

## 2. Materials and Methods

### 2.1. Preparation of Samples

**Figure 1:**
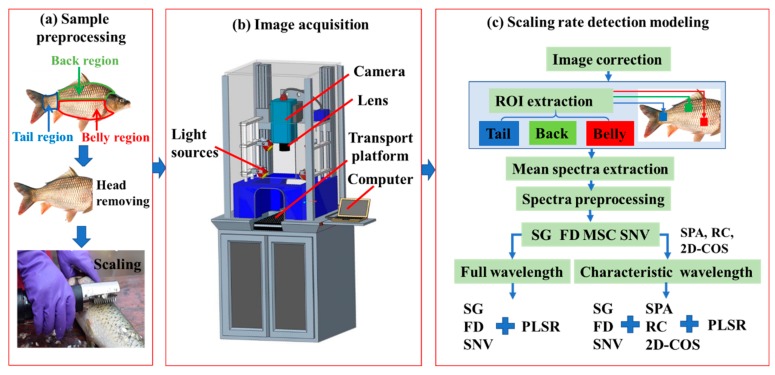
Experimental procedure. (ROI: region of interest, SG: Savitzky–Golay, FD: first derivative, MSC: multiple scattering correction, SNV: standard normal variate, SPA: successive projection algorithm, RC: regression coefficient, 2D-COS: two-dimensional correlation spectroscopy, PLSR: partial least square regression.)

In this study, fresh carp were used as the experimental samples. The experimental procedure is shown in Figure 1. The fresh carp were bought from the local market of Dalian, China. The fish heads were removed first to save scanning time (Figure 1a). Then, the samples were scaled using a handheld scale remover (Lijiu Industrial Co. Ltd., Zheng Zhou, China). The scales and dirt were cleaned, and then the surface water was absorbed using absorbent paper. Hyperspectral information was captured by the hyperspectral imaging system (Figure 1b). The sample body was divided into three regions (Figure 1a) for analysis because the response of the carp back, belly, and tail regions was quite different in spectra and images. After hyperspectral data acquisition and spectral correction, a rectangular area (2 × 2 cm^2^) within the hyperspectral images of the belly, back, and tail regions was selected and identified as the region of interest (ROI) for extracting hyperspectral information (Figure 1c).

A total of 50 carp were scaled to different degrees in different regions (back, belly, and tail) on either side of the fish body, resulting in 100 samples being used to model for each region. The image processing method realized by Photoshop CS 6 (Adobe Systems Inc. San Jose, CA, USA) was used to measure the pixel area of the relevant region. The measured value of scaling rate (*P*) was calculated as follows:

(1) $$P=\frac{P1}{P2}\times 100\%$$

where *P*1 (pixel^2^) is the scaling pixel area in the specific region (back, belly or tail) and *P*2 (pixel^2^) is the whole fish pixel area of the specific region (back, belly or tail). The maximum (max), minimum (min), and standard deviation (SD) of the scaling rate in the different regions of the samples for modeling are shown in Table 1.

### 2.2. Hyperspectral Imaging System

Hyperspectral data were captured using the GaiaField-V10 hyperspectral imaging system (Dualix Spectral Image Technology Co. Ltd., Sichuan, China). Figure 1b shows the core components of the system, which includes a computer, a spectral camera, a lens, light sources, and a transport platform. The spectral acquisition range of the system was 387.1–1024.7 nm. The speed of the conveyor belt was set to 0.9 cm/s. The object distance and exposure time were set to 30 cm and 22 m/s, respectively. All measurements were conducted in triplicate for each sample.

### 2.3. Correction of Spectra

The original hyperspectral images were corrected to the reflection pattern to diminish the influence of illumination and system sensitivity and reduce the difference between the camera and the physical configuration of the imaging system. A rectangular area of the white standard correction board in the collected image was selected as the white reference image. The lens was covered with a cap, the light source was closed, and then the dark reference image was captured [32,33]. The corrected image was calculated as follows:

(2) $$R_{c}=\frac{I_{R}-D}{I_{W}-D}\times 100\%$$

where *I_R_* is the original uncorrected hyperspectral image, *I_W_* is a white reference image, *D* is a dark reference image, and *R_C_* is the corrected image.

### 2.4. Spectral Preprocessing

Preprocessing of the original spectral data is required to eliminate noise, remove co-linear or improper information, and enhance the performance of the models [34]. In this study, three popular preprocessing methods were applied before modeling: SG, FD, MSC, and SNV. SG smoothing, also known as polynomial smoothing, is a polynomial method based on the least squares fitting. SG can eliminate high-frequency random errors and improve the signal-to-noise ratio to some extent [35]. The polynomial order and the number of window points were set to 3 and 7, respectively. FD can eliminate the overlapping of spectra caused by the background interference and baseline shift, and present a clearer spectral contour with obvious peaks and valleys, which is widely used to enhance the characteristics of spectra [25]. The FD of wavelength *W* was calculated as follows:

(3) $${\mathrm{FD}}_{\lambda \left(W\right)\;}=\frac{R_{\lambda \left(W+1\right)}+R_{\lambda \left(W-1\right)}}{\Delta \lambda }$$

where ${\mathrm{FD}}_{\lambda \left(W\right)}$ represents the FD transformation at a specific wavelength (W) midpoint between wavelengths W + 1 and W − 1; $R_{\lambda \left(W+1\right)}$ is the reflectance value of wavelength W + 1, $R_{\lambda \left(W-1\right)}$ is the reflectance value of wavelength W − 1, Δλ is the difference between wavelength W + 1 and wavelength W − 1. MSC has the ability to eliminate the influence of baseline effect and some multiplicative scattering and transform spectra more closely to the reference spectrum [36]. In this study, MSC was mainly applied to eliminate the relative baseline shift, multiplicative scattering, and the migration between spectra. Therefore, the average spectrum of the whole spectra data set in each region was selected as the reference spectrum, and the corrected spectrum (*X_MSC_*) was calculated as follows:

(4) $$\bar{X_{i}}=\overset{n}{\underset{i=1}{\sum }}\frac{X_{i}}{n}$$

(5) $$X_{i}=m_{i}{\bar{X}}_{i}+b_{i}$$

(6) $$X_{MSC}=\frac{\left(X_{i}-b_{i}\right)}{m_{i}}$$

where $X_{i}\;$ represents the spectrum of a sample; *n* is the number of the samples in each region; $\bar{X_{i}}$ represents a reference spectrum, which is the average spectrum of the whole data set in this paper; the estimates of $m_{i}$ and $b_{i}$ can be calculated through ordinary least squares regression of $X_{i}$ onto $\bar{X_{i}}$. SNV also is a famous pretreatment that can effectively remove light-scattering effects. It is a row-oriented transformation, which could eliminate the scattering effect without changing the original spectral shape [37]. The calculation process is as follows:

(7) $$SNV=\frac{x-\bar{x}}{\sqrt{\frac{\sum_{k=1}^{m}{\left(x_{k}-\bar{x}\right)}^{2}}{m-1}}}$$

where *x* is the mean spectral reflectivity for each wavelength within each carp sample, and $\bar{x}=\frac{\sum_{k=1}^{m}x_{k}}{m}$, *m* represents the number of wavelengths, and *k* = 1, 2, ……, *m*.

### 2.5. Selection of CWs

Considering the large amount of hyperspectral data and redundant information, it is very important to implement the dimensionality reduction of spectra. SPA, 2D-COS, and RC were utilized for selecting CWs in this study.

SPA is an effective wavelength dimension reduction method with minimum redundancy and collinearity using the projection analysis of vectors [38]. The mean spectral reflectivity of all wavelengths was used for SPA. The minimum number of wavelengths corresponding to the minimum root mean square error (*RMSE*) was selected as the number of CWs.

RC (β-coefficients), as a commonly used method of CW selection, often obtains a good performance combined with PLSR. The basic principle of the algorithm is to calculate RC by building a PLSR model using FW. According to the absolute values of RC, the CWs were selected. The peaks or valleys corresponding to the highest absolute values in RC plots were selected as optimal wavelengths [39].

Another very versatile spectral analytical technique called 2D-COS can be used for sorting out key information in a broad range of spectroscopic signals such as Raman spectrum, nuclear magnetism, and UV-vis-NIR spectrum, among others [40]. In 2D-COS analysis, the raw spectral data were generated into synchronous and asynchronous correlation orthogonal representations. In the synchronous spectrum map, the auto-peaks located at the diagonal line represent the varying intensity of the system induced by the external perturbation, which could be used to characterize the variations of spectral intensities at corresponding wavelengths. As long as the spectral intensity changes dramatically enough, the auto-peak will appear at a certain wavelength. In this study, the external perturbation is scaling rate, and the auto-peak of synchronous spectrum was introduced to recognize the CWs.

### 2.6. PLSR Scaling Rate Detection Model

PLSR is an effective multivariate statistical method for analyzing the linear relationships between multiple independent and dependent variables. It is superior to the general linear regression method, especially in the highly correlated continuous process [41]. In the present study, the PLSR scaling rate detection model for carp was established based on FWs and CWs. The calibration and prediction sets were formed using 60 and 40 samples with different scaling rates, respectively. The mean spectral reflectivity was used as the input, and the scaling rate was used as the output for modeling. The coefficient of determination in calibration and prediction set (*R_C_*^2^ and *R_P_*^2^), root mean square error by calibration (*RMSEC*), root mean square error by prediction (*RMSEP*), and relative percent deviation (*RPD*) were used for evaluating the performance of the model [42]. Generally speaking, a well-performing model should have high *R^2^* and *RPD* values, low *RMSEC* and *RMSEP* values, and small differences between them [43]. A value of *R_P_*^2^ lower than 82% indicates that the performance of the model is inaccurate and relatively poor; between 82% and 90% indicates the model performance is good and available for practical application; and higher than 90% indicates an excellent accuracy of the model [44]. A value of *RPD* greater than 2 indicates that the model possesses an excellent prediction ability; between 1.50 and 2 indicates that the model can be used for detection; and less than 1.5 indicates that the model cannot be used [45].

All the collected spectral data were processed and analyzed in the image processing toolbox of image visualization software ENVI5.1 (Research Systems Inc., Solutions, Boulder, CO, USA) and MATLAB 2012a (Math Works Inc., Natick, MA, USA).

### 2.7. Scanning Electron Microscopy (SEM)

The microstructure of scale and skin of carp samples were characterized using SEM. After the sample was frozen and fixed at −80 °C, it was moved to a vacuum freeze-dryer for drying, then mounted on a metal stub, coated with Pd, and imaged by a JSM-7800F SEM (JEOL Ltd., Tokyo, Japan) which operated at 10 kV.

## 3. Results and Discussion

### 3.1. Data Analysis of Original Spectra

**Figure 2:**
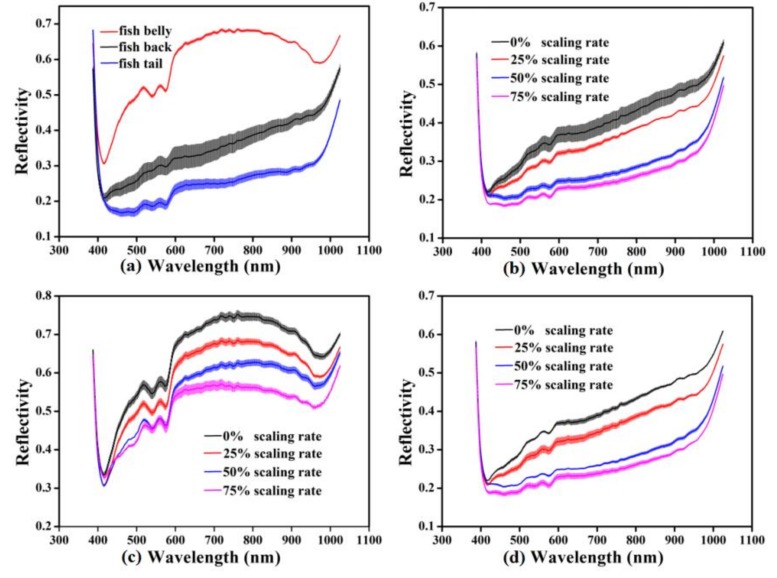
Original mean spectra with SD: (**a**) mean spectra of the back, belly, and tail regions without scaling; (**b**) mean spectra of the back region with different scaling rate; (**c**) mean spectra of the belly region with different scaling rate; (**d**) mean spectra of the tail region with different scaling rate.

**Figure 3:**
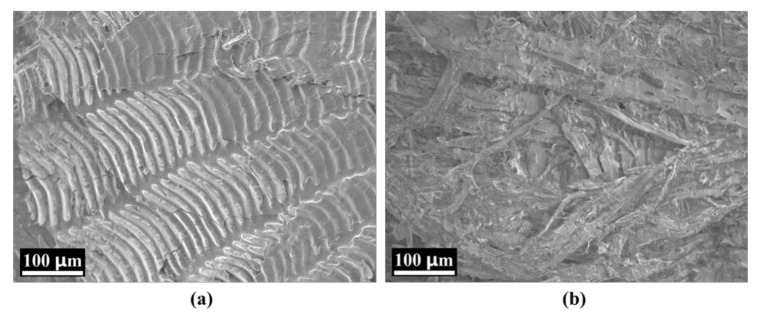
Scanning electron microscopy (SEM) images of (**a**) carp scale and (**b**) carp skin.

The original mean spectra with SD of the different sample regions (back, belly, and tail) possessing different scaling rates are plotted in Figure 2. They were obtained by averaging the reflectance values of all the pixels in the ROI at the spectral range of 387.1–1024.7 nm. Figure 2a shows the mean spectra of the belly, back, and tail regions without scaling, displaying the successive increase in spectral reflectivity. The spectral reflectivity of the back and tail increased steadily around 600–980 nm, which increased and then decreased for the belly region. Figure 2b–d shows the original mean spectra of the different regions with different scaling rates, respectively. All illustrated an obvious absorption peak around 420 nm, which might be caused by the absorption of porphyrin compounds in the visible region [46]. Furthermore, several absorption peaks were observed in the range of 500–600 nm, which might be due to the existence of metmyoglobin [47]. The absorption peak around 960 nm might be related to the absorption vibration of water [48]. The absorption and reflection of light for fish scales and skin are different because of the differences in their structure and morphology, resulting in different spectral information with different scaling rates. Figure 2 shows that the spectral reflectivity decreased with the increase in the scaling rate. It illustrates that the spectral reflectivity of fish scales was higher than that of skin in the range of 387.1–1024.7 nm. Hence, the more the scales were removed, the more the visible or near-infrared light was absorbed by the skin of the carp samples. The variation in mean spectra clearly demonstrated that the spectral reflectivity was significantly affected by different areas of carp skin and scales, i.e., the scaling rate. In view of this, the microstructure of the scales and skin of the carp samples were examined using SEM. As shown in Figure 3, since the scale is mainly composed of collagen, scleroprotein, hydroxyapatite and calcium phosphate [49], its SEM image presents a neat inorganic fiber-board structure (Figure 3a). However, Figure 3b reflects an obvious staggered myofiber structure. It illustrated significant differences in the microstructure between the scale and skin of carp due to their different composition. This is consistent with the different responses of the mean spectra illustrated in Figure 2. Therefore, it was feasible to divide the carp sample into three different regions for analysis and extract the mean spectral reflectivity of ROI for detection.

### 3.2. Spectral Preprocessing

**Figure 4:**
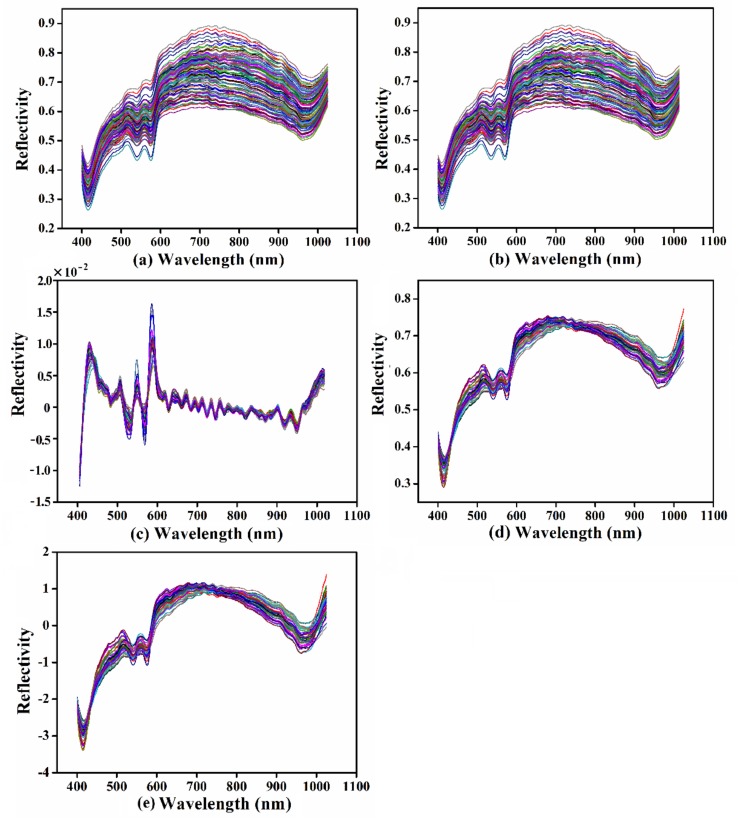
Mean spectra of carp with different preprocessing methods: (**a**) original mean spectra; (**b**) mean spectra preprocessed by SG; (**c**) mean spectra preprocessed by FD; (**d**) mean spectra preprocessed by MSC; and (**e**) mean spectra preprocessed by SNV.

Taking the belly region as an example, different preprocessing methods were analyzed. The original and preprocessed spectra of the belly are presented in Figure 4. Only the spectral wavelengths that ranged from 400 nm to 1024.7 nm were applied for subsequent analysis (352 wavelengths) owing to the obvious overlapping parts of the spectra at the range of 387.1–399.8 nm. As shown in Figure 4b, the spectra were smoothed through SG, and the effects of noise caused by relevant experimental factors were filtered out, which may help to improve the accuracy and robustness of the model [50]. In Figure 4c, the mixed spectra were decomposed by FD, and the peaks and valleys of spectral curves became more obvious. Many studies have proven the potential of spectra preprocessed by FD for estimating physicochemical indexes [25]. Figure 4d,e showed that the scattering information was reduced by MSC and SNV, respectively. There was no significant difference between the two methods. However, the spectrum after MSC was closer to the reference spectrum, while the shape of the spectrum after the SNV was closer to the original spectrum. The spectra preprocessed by SG, MSC, and SNV were used to establish the PLSR scaling rate detection model, and the optimal pretreatment was determined based on the modeling results.

### 3.3. Selection of CWs

#### 3.3.1. SPA

In this study, SPA was used to realize data dimension reduction and select the CWs with the useful information for scaling rate detection. The SPA results of the optimal CWs are shown in Table 2. These CWs basically covered the whole spectral data range between 400–1024.7 nm, which eliminated the redundant information and might represent the most useful information in the FWs. As shown in Table 2, the distribution of the selected CWs might be related to the differences in color and protein and moisture contents between the scale and skin of carp. The selected wavelengths of 983.8, 993.0, 989.3, and 970.8 nm (near 960 nm) were attributed to water absorption bands. The selected wavelengths around 700 nm were due to C-H stretching overtones and protein interactions. Moreover, the other selected wavelengths were consistent with the tendency of higher scaling rate demonstrating lower reflectivity as described in Section 3.1, leading to a good effect on the performance of the PLSR model.

#### 3.3.2. RC

**Figure 5:**
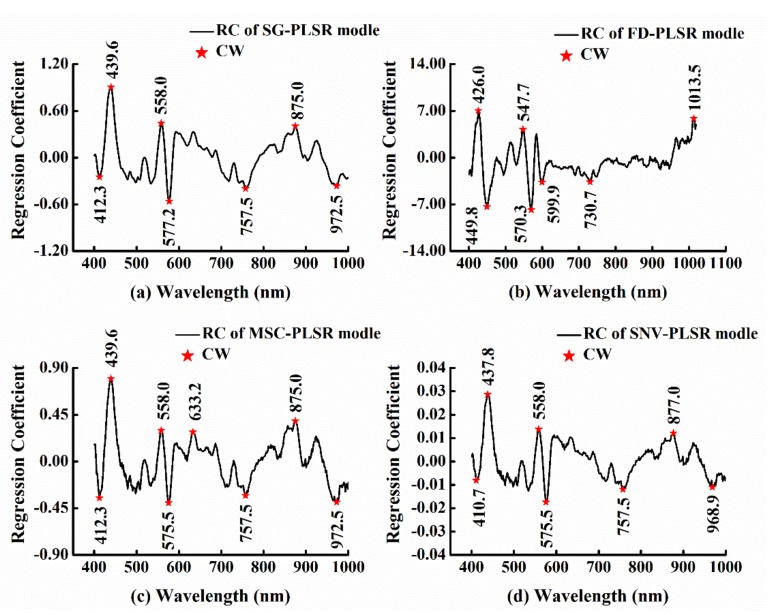
CWs of the belly region selected by the method of RC based on different preprocessed methods (**a**) SG-FD, (**b**) FD, (**c**) MSC, and (**d**) SNV.

Taking the belly region as an example, the CWs selected by the method of RC based on different preprocessed methods of SG, FD, MSC, and SNV are shown in Figure 5. As can be seen from the plots, the wavelengths corresponding to the RC with a large absolute value were selected as the CWs. As shown in Table 3, the CWs selected by RC were wider than those selected by SPA, which may be mainly ascribed to C-H stretching overtones, protein interaction, and water absorption. It was similar to the CWs used in Huang’s study (440 nm, 497 nm, 535 nm, 574 nm, 728 nm, 910 nm, 955 nm and 1022 nm) [28].

#### 3.3.3. 2D-COS

**Figure 6:**
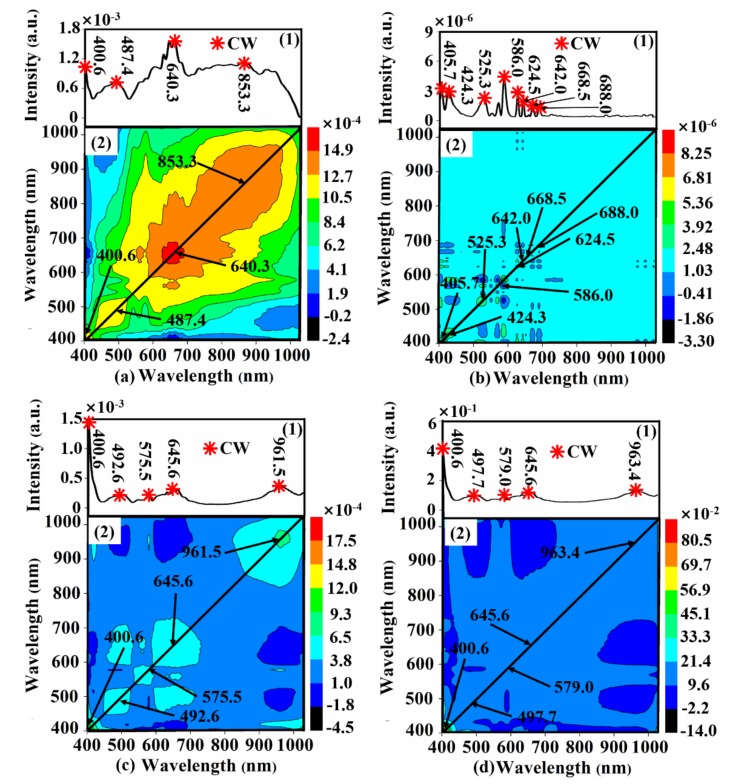
The 2D-COS spectrum maps of the belly region, which were preprocessed by (**a**) SG, (**b**) FD, (**c**) MSC, and (**d**) SNV, respectively.

Taking the belly region spectra preprocessed by SG as an example, the 2D-COS spectrum maps of samples with various scaling rates based on different preprocessing methods (SG, FD, MSC, and SNV) are shown in Figure 6. There was one distinct auto-peak corresponding to the wavelength of 640.3 nm illustrated on the diagonal line in synchronous spectra (Figure 6(a2)), and three weak auto-peaks appeared in corresponding synchronous spectra (Figure 6(a1)), resulting in four CWs of 400.6 nm, 487.4 nm, 640.3 nm and 853.3 nm. Accordingly, the CWs with different preprocessed methods of different regions were determined and shown in Table 4, respectively. The number of CWs reduced by 2D-COS is lower than that of the above two methods (SPA and RC), which were also distributed around the part of the CWs selected by SPA and RC.

### 3.4. Analysis of the PLSR Scaling Rate Detection Model

The PLSR scaling rate detection model of carp back, belly, and tail was established using spectra of FWs and CWs, resulting in models named SG-PLSR, FD-PLSR, MSC-PLSR, SNV-PLSR, SG-SPA-PLSR, FD-SPA-PLSR, MSC-SPA-PLSR, SNV-SPA-PLSR, SG-RC-PLSR, FD-RC-PLSR, MSC-RC-PLSR, SNV-RC-PLSR, SG-2D-COS-PLSR, FD-2D-COS-PLSR, MSC-2D-COS-PLSR, and SNV-2D-COS-PLSR, respectively.

#### 3.4.1. Model of the Back Region

As shown in Table 5, the *R_C_*^2^ and *R_P_*^2^ of models using FWs (SG-PLSR, FD-PLSR, MSC-PLSR, and SNV-PLSR) were all higher than 90%. It showed that the models had excellent fitting degree. *RPD* values were all greater than two, indicating a strong predictive ability for back scaling rate with full-range spectra. The absolute differences between *RMSEC* and *RMSEP* of SG-PLSR, FD-PLSR, MSC-PLSR, and SNV-PLSR models were 1.37%, 0.39%, 0.79%, and 0.92%, respectively, indicating that the models using FWs had strong stability. However, the variable number of models using FW spectra was 352 (Table 5), which might take a lot of time to compute. Therefore, the models using CWs were established, and the *R_C_*^2^ and *R_P_*^2^ of the models all decreased. Of these, the models using 2D-COS had a big drop, whereas the models using SPA and RC had a slight drop and a good fitting degree. It indicated that SPA and RC could reduce the data dimension effectively and had little effect on the prediction result integrating with different pretreatments (SG, FD, MSC, and SNV). Considering the better modeling performance and fewer variables, the FD-RC-PLSR model was recommended for further implementation in scaling rate detection in the back region of carp.

#### 3.4.2. Model of the Belly Region

As shown in Table 6, the *R_C_*^2^ and *R_P_*^2^ of detection models using FWs (SG-PLSR, FD-PLSR, MSC-PLSR, and SNV-PLSR) were all higher than 90%, indicating the excellent fitting degree. Of these, *RMSEC* and *RMSEP* had small differences, and *RPD* were all larger than two, indicating that the models were highly stable and had excellent prediction ability. The SNV-PLSR model possessed the best detection precision and robustness with the highest *R_C_*^2^, *R_P_*^2^, and *RPD* values. However, the large variable numbers (352) might take a lot of time on calculation in actual detection. Comparing the performances of the models based on different CW methods (Table 6), the models using SPA and RC had better results. As shown in the results of 2D-COS-PLSR model, the CWs preprocessed by FD resulted in a good precision for scaling rate detection of the belly region. It indicated that the combination of FD with 2D-COS could obtain better prediction effect in some cases. According to the values of *R_C_*^2^ and *R_P_*^2^, and the differences between *RMSEC* and *RMSEP*, the model stability and accuracy using SPA is obviously better than the others. Considering the evaluating parameters of PLSR models using CWs, the SNV-SPA-PLSR model had the highest *R_C_*^2^ (93.44%) and *R_P_*^2^ (90.81%), the smallest absolute difference between *RMSEC* and *RMSEP* (0.87%), and *RPD* >2, which was proved to be the optimized PLSR model for scaling rate detection in the belly region.

#### 3.4.3. Model of the Tail Region

The model evaluating parameters of the carp tail region using preprocessed FWs and CWs are shown in Table 7. The *R_C_*^2^ of SG-PLSR, FD-PLSR, MSC-PLSR, and SNV-PLSR models, that is, the models using FW spectra, were all higher than or equal to 98%, with a very small decrease in *R_P_*^2^. All the models using FWs had low *RMSEC* and *RMSEP* values as well as small differences between them. Besides, all the *RPD* values of the FW model were greater than two. This indicated that the model using FWs had high accuracy, robustness, and prediction ability. The SPA, RC, and 2D-COS methods were also used for modeling due to more input variables in modeling using FWs. Generally, the CW selection methods can be used to simplify the huge amount of hyperspectral data and explore an online multispectral imaging system. However, they may also result in ignoring or losing useful information and detail. As shown in Table 7, the performance of the model decreased as the number of wavelength used in modeling decreased, especially for the 2D-COM-PLSR model. Nevertheless, the model using SPA and RC still presents with good performance. Due to the small difference in prediction ability and robustness of models using SPA and RC, SNV-RC-PLSR had the highest accuracy (*R_C_*^2^ = 95.34% and *R_P_*^2^ = 93.71%), and the PLSR model integrated with SNV and RC was determined to be more conducive to detecting scaling rate in the carp tail, resulting in an optimal model of SNV-RC-PLSR.

**Figure 7:**
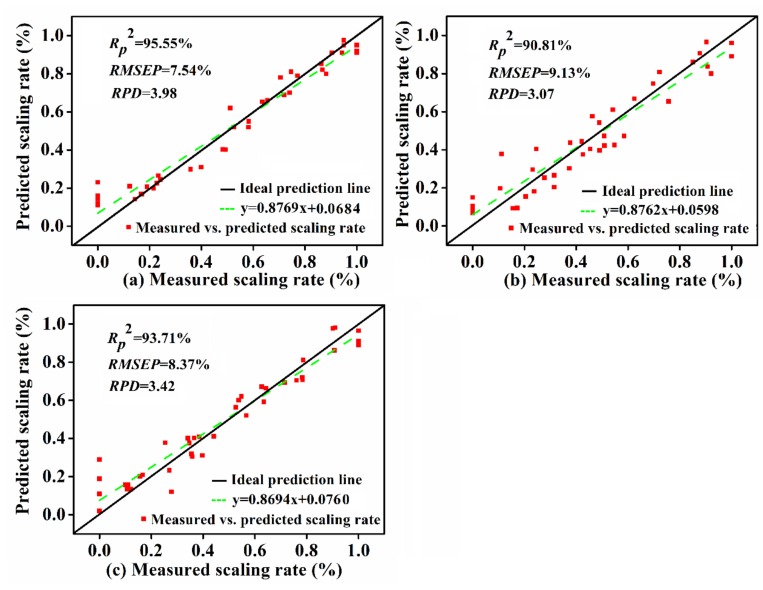
Measured scaling rate vs. predicted scaling rate by the optimal model in (**a**) back, (**b**) belly, and (**c**) tail regions of carp in the prediction set.

To sum up, the FD-RC-PLSR model for the back, SNV-SPA-PLSR model for the belly, and SNV-RC-PLSR model for the tail region were determined to be the optimized models, and the performance of the prediction set is shown in Figure 7, respectively. The estimated scaling rates in the prediction set of the optimized models were in line with the measured scaling rates, as the data points mostly located near the ideal prediction line, which indicated that the PLSR model combined with an appropriate pretreatment and the specific dimension reduction method presented a stronger precision and robustness for the prediction of the scaling rate of carp.

## 4. Conclusions

In this study, hyperspectral technology was used to investigate the potential of nondestructive detection of the scaling rate of carp. The carp samples were divided into three different regions (back, belly, and tail) for the analysis. SG, FD, MSC, and SNV were employed for spectral preprocessing. SPA, RC, and 2D-COS were used to select CWs. The PLSR scaling rate detection models of different regions were established based on the FWs and CWs. After a comparison of the performances of the models, the FD-RC-PLSR model based on eight CWs (422.6 nm, 525.3 nm, 568.6 nm, 691.5 nm, 736.0 nm, 882.4 nm, 983.8 nm, and 996.8 nm), SNV-SPA-PLSR model based on eight CWs (402.3 nm, 437.9 nm, 478.8 nm, 544.3 nm, 608.7 nm, 746.8 nm, 875.1 nm, and 989.3 nm), and SNV-RC-PLSR model based on eight CWs (412.3 nm, 437.8 nm, 497.7 nm, 559.9 nm, 575.5 nm, 594.7 nm, 764.7 nm, and 952.2 nm) were respectively recommended as the optimized model for predicting the scaling rate in the back, belly, and tail regions. This study showed that hyperspectral technology was a promising method to predict the scaling rate of carp. Because hyperspectral data is band limited, and the characteristics of carp scales and skin are special, the scaling regions are easily aliased during visualization. If sampled at or above its Nyquist rate, the aliasing may be avoided. Therefore, based on the optimal CWs and models, oversampling and image processing techniques would be required to realize visualization of the scaling region.

## Figures and Tables

**Table 1:** The statistical data of the scaling rate in different regions of the samples. SD: standard deviation.

Different Regions	Number of Samples	Max (%)	Min (%)	SD
Back	100	100.00	0.00	0.23
Belly	100	100.00	0.00	0.22
Tail	100	100.00	0.00	0.24

**Table 2:** Characteristic wavelengths (CWs) selected by SPA.

Different Regions	Preprocessing Method	Number of CWs	CWs (nm)
Back	SG	5	400.6, 497.7, 568.6, 663.2, and 716.4
	DF	5	572.0, 635.0, 693.3, 771.9, and 983.8
	MSC	6	400.6, 419.2, 497.7, 528.7, 645.6, and 700.4
	SNV	5	400.6, 460, 520.1, 579, and 764.7
Belly	SG	7	400.6, 437.9, 473.7, 613.9, 759.3, 875.1, and 993.0
	DF	7	489.1, 551.2, 540.8, 605.2, 636.8, 766.5, and 902.6
	MSC	6	400.6, 521.8, 556.4, 577.3, 608.7, 961.5
	SNV	8	402.3, 437.9, 478.8, 544.3, 608.7, 746.8, 875.1, and 989.3
Tail	SG	5	415.8, 437.9, 499.4, 521.8, and 573.8
	DF	7	412.4, 478.8, 511.5, 566.8, 768.3, 970.8, and 1015.4
	MSC	5	415.8, 436.2, 497.7, 520.1, and 682.7
	SNV	5	407.4, 431.1, 497.7, 656.1, and 847.8

**Table 3:** CWs selected by RC.

Different Regions	Preprocessing Method	Number of CWs	CWs (nm)
Back	SG	4	470.2, 575.5, 645.5, and 761.0
	DF	8	422.6, 525.3, 568.6, 691.5, 736.0, 882.4, 983.8, and 996.8
	MSC	7	414.1, 502.8, 573.7, 645.5, 759.2, 875.0, and 970.7
	SNV	4	446.3, 575.5, 718.2, and 762.9
Belly	SG	7	412.3, 439.6, 558.0, 577.2, 757.5, 875.0, and 972.5
	DF	7	426.0, 449.8, 547.7, 570.3, 599.9, 730.7, and 1013.5
	MSC	8	412.3, 439.6, 558.0, 575.5, 633.2, 757.5, 875.0, and 972.5
	SNV	7	410.7, 437.8, 558.0, 575.5, 757.5, 877.0, and 968.9
Tail	SG	8	412.3, 437.8, 497.7, 556.4, 577.2, 594.7, 762.9, and 959.7
	DF	9	407.4, 424.3, 449.8, 568.6, 582.5, 596.4, 750.3, 983.8, and 1011.7
	MSC	7	412.3, 437.8, 499.3, 559.9, 577.2, 594.7, and 764.77
	SNV	8	412.3, 437.8, 497.7, 559.9, 575.5, 594.7, 764.7, and 952.2

**Table 4:** CWs by 2D-COS.

Different Regions	Preprocessing Method	Numbers of CWs	CWs (nm)
Back	SG	3	400.6, 656.1 and 855.1
	DF	3	405.7, 422.6, and 624.5
	MSC	3	400.6, 661.4, and 887.9
	SNV	3	400.6, 645.6, and 893.4
Belly	SG	4	400.6, 487.4, 640.3, and 853.3
	DF	8	405.7, 424.3, 525.3, 586.0, 624.5, 642.0, 668.5, and 688.0
	MSC	5	400.6, 492.6, 575.5, 645.6, and 961.5
	SNV	5	400.6, 497.7, 579.0, 645.6, and 963.4
Tail	SG	3	551.5, 552.9, and 612.2
	DF	5	400.6, 427.7, 525.3, 568.6, and 584.2
	MSC	4	415.8, 508.0, 617,4, and 961.5
	SNV	4	417.5, 473.7, 605.2, and 965.2

**Table 5:** Evaluating parameters of the PLSR model of the back region.

Model	No.	Calibration Set				Prediction Set				
		*R_C_*^2^ (%)	*RMSEC* (%)	SD_C_	SE_C_	*R_P_*^2^ (%)	*RMSEP* (%)	*RPD*	SD_P_	SE_P_
SG-PLSR	352	98.92	1.96	0.19	0.02	96.85	3.33	5.41	0.18	0.03
FD-PLSR	352	99.06	1.82	0.18	0.03	99.72	1.43	12.59	0.18	0.03
MSC-PLSR	352	99.64	1.63	0.18	0.02	98.73	2.42	7.44	0.18	0.03
SNV-PLSR	352	99.43	1.74	0.19	0.02	98.16	2.66	7.14	0.19	0.03
SG- SPA -PLSR	5	90.63	6.36	0.21	0.03	88.23	7.19	2.64	0.19	0.03
DF-SPA -PLSR	5	88.92	9.87	0.26	0.03	86.93	10.59	2.46	0.26	0.03
MSC-SPA -PLSR	6	87.21	6.87	0.18	0.02	83.13	7.62	1.84	0.14	0.02
SNV-SPA -PLSR	5	95.07	6.72	0.25	0.03	93.23	8.13	3.44	0.28	0.04
SG-RC-PLSR	4	93.04	8.02	0.25	0.03	91.19	10.24	2.79	0.29	0.05
FD-RC-PLSR	8	96.23	6.20	0.26	0.03	95.55	7.54	3.98	0.30	0.05
MSC-RC -PLSR	7	89.01	9.81	0.26	0.03	88.76	11.79	2.40	0.28	0.04
SNV-RC -PLSR	4	92.82	8.34	0.25	0.03	91.17	10.07	3.01	0.30	0.05
SG-2D-COS -PLSR	3	44.42	24.60	0.27	0.03	42.57	26.71	1.09	0.29	0.05
FD-2D-COS -PLSR	3	43.60	24.71	0.27	0.03	44.24	26.35	1.02	0.27	0.03
MSC-2D-COS -PLSR	3	44.66	21.96	0.23	0.03	41.72	26.90	1.13	0.30	0.05
SNV-2D-COS -PLSR	3	45.31	21.83	0.21	0.03	40.46	25.81	0.85	0.22	0.03

No.: the variable number of models. SD_C_ and SD_P_: Standard deviation of the predicted scaling rate of calibration set and prediction set. SE_C_ and SE_P_: Standard error of the predicted scaling rate of calibration set and prediction set.

**Table 6:** Evaluating parameters of PLSR model of the belly region.

Model	No.	Calibration Set					Prediction Set				
		*R_C_*^2^ (%)	*RMSEC* (%)		SD_C_	SE_C_	*R_P_*^2^ (%)	*RMSEP* (%)	*RPD*	SD_P_	SE_P_
**SG-PLSR**	352	94.01	4.82	0.17		0.02	91.06	5.83	2.92	0.17	0.03
DF-PLSR	352	97.32	3.32	0.17		0.02	96.67	6.54	2.56	0.17	0.03
MSC-PLSR	352	93.42	4.94	0.17		0.02	91.20	5.77	2.77	0.16	0.03
SNV-PLSR	352	99.33	1.63	0.27		0.04	98.34	2.54	10.24	0.26	0.04
SG-SPA -PLSR	7	92.43	5.46	0.19		0.02	89.33	6.44	2.80	0.18	0.03
DF-SPA -PLSR	7	92.13	8.98	0.25		0.03	91.19	8.95	2.79	0.25	0.04
MSC-SPA -PLSR	6	91.23	6.26	0.16		0.02	90.21	7.02	2.27	0.16	0.03
SNV-SPA -PLSR	8	93.44	8.05	0.26		0.03	90.81	9.13	3.07	0.28	0.04
SG-RC -PLSR	7	90.18	9.76	0.25		0.03	86.07	12.19	2.05	0.25	0.04
DF-RC -PLSR	7	90.62	9.61	0.25		0.03	87.66	10.58	2.36	0.25	0.04
MSC-RC -PLSR	8	91.56	8.75	0.27		0.03	88.51	10.68	2.43	0.26	0.04
SNV-RC -PLSR	7	89.06	9.92	0.26		0.03	85.72	11.56	1.73	0.20	0.03
SG-2D-COS -PLSR	4	50.50	20.83	0.19		0.02	47.28	24.28	0.91	0.22	0.03
DF-2D-COS -PLSR	8	91.24	9.29	0.25		0.03	87.72	10.57	2.37	0.25	0.04
MSC-2D-COS -PLSR	5	62.96	18.68	0.20		0.03	60.64	19.17	1.10	0.21	0.03
SNV-2D-COS -PLSR	5	58.99	19.71	0.23		0.03	55.61	20.33	1.18	0.24	0.04

No.: the variable number of models. SD_C_ and SD_P_: Standard deviation of the predicted scaling rate of calibration set and prediction set. SE_C_ and SE_P_: Standard error of the predicted scaling rate of calibration set and prediction set.

**Table 7:** Evaluating parameters of PLSR model of the tail region.

Model	No.	Calibration Set				Prediction Set				
		*R_C_*^2^ (%)	*RMSEC* (%)	SD_C_	SE_C_	*R_P_*^2^ (%)	*RMSEP* (%)	*RPD*	SD_P_	SE_P_
SG-PLSR	352	97.92	2.82	0.20	0.03	96.21	4.32	4.86	0.21	0.03
DF-PLSR	352	98.34	1.58	0.20	0.03	98.11	2.34	8.55	0.20	0.03
MSC-PLSR	352	98.31	1.53	0.20	0.03	97.52	4.07	4.91	0.20	0.03
SNV-PLSR	352	97.82	1.76	0.24	0.03	96.15	3.22	7.45	0.24	0.04
SG-SPA -PLSR	5	92.16	6.38	0.24	0.03	89.27	9.45	2.54	0.24	0.04
DF-SPA -PLSR	7	94.78	7.09	0.24	0.03	93.30	8.40	2.86	0.24	0.04
MSC-SPA -PLSR	5	94.56	7.76	0.25	0.03	91.38	9.95	2.41	0.24	0.04
SNV-SPA -PLSR	5	89.69	8.96	0.27	0.04	87.22	12.22	2.29	0.28	0.04
SG-RC -PLSR	8	93.72	7.75	0.25	0.03	92.89	9.76	2.67	0.26	0.04
DF-RC -PLSR	9	91.43	9.83	0.25	0.03	89.52	10.86	2.30	0.25	0.04
MSC-RC -PLSR	7	90.90	8.80	0.25	0.03	89.86	11.02	2.40	0.26	0.04
SNV-RC -PLSR	8	95.34	6.66	0.25	0.03	93.71	8.37	3.42	0.29	0.05
SG-2D-COS -PLSR	3	54.64	19.84	0.24	0.03	53.05	21.65	1.12	0.24	0.04
DF-2D-COS -PLSR	5	52.62	20.65	0.23	0.03	50.71	22.49	1.02	0.23	0.04
MSC-2D-COS-PLSR	4	63.81	18.55	0.23	0.03	59.71	20.12	1.25	0.25	0.04
SNV-2D-COS -PLSR	4	63.53	17.48	0.21	0.03	59.88	20.02	1.16	0.23	0.04

No.: the variable number of models. SD_C_ and SD_P_: Standard deviation of the predicted scaling rate of calibration set and prediction set. SE_C_ and SE_P_: Standard error of the predicted scaling rate of calibration set and prediction set.
